# An Exploratory Investigation of the Influence of Setting and Meaning on Emotion Responses to Virtual Reality Environments

**DOI:** 10.3390/bs16071195

**Published:** 2026-07-15

**Authors:** David Anthony Redmond, Brendan Rooney, Pamela Gallagher

**Affiliations:** 1 School of Psychology, Dublin City University, Glasneven Campus, Dublin 9, D09 V209 Dublin, Ireland; david.redmond28@mail.dcu.ie (D.A.R.); pamela.gallagher@dcu.ie (P.G.); 2 School of Psychology, University College Dublin, Dublin 4, D04 F6X4 Dublin, Ireland

**Keywords:** virtual reality, emotion, positive technology, experimental design

## Abstract

The route to wellbeing is often divided into hedonic (pleasure or relaxation) and eudaimonic (meaning or growth) pathways. Positive technology is a growing research area which aims to use technology to facilitate engagement in wellbeing-supporting activities. While virtual reality (VR) is increasingly used to support day-to-day wellbeing, the mechanisms underlying these effects remain unclear and VR research typically determines success based on outcomes. This leaves a gap whereby the processes which facilitate outcomes are less understood. This exploratory study examined the relative effects, on participant emotion responses (*N* = 35), of hedonic (nature vs. urban setting) and eudaimonic (personally meaningful vs. not personally meaningful) VR environments. Results showed that the level of personal meaning associated with an environment influenced emotion outcomes and visual setting did not. Notably, meaningful environments elicited a “mixed-emotional” state, increasing both positive and negative emotional responses. The results highlight the potential for short, personalised virtual experiences to elicit emotionally complex responses that are theoretically consistent with eudaimonic processes like meaningful reminiscence. Results are discussed in relation to the relative influence of hedonic and eudaimonic stimuli to be explored in future research that builds on these findings.

## 1. Introduction

Positive technology is the field of study exploring the use of technology systems to support individual wellbeing (Gaggioli et al., 2017; Inghilleri et al., 2015; Riva et al., 2012). Virtual reality (VR) has been identified as a promising positive technology tool due to its ability to immerse the participant in a specially designed environment and create specific, ecologically valid scenarios (Gaggioli et al., 2017; Parsons, 2015; Riva et al., 2012; Riva & Gaggioli, 2015). Studies have shown VR has efficacy, particularly in positively influencing state-based hedonic wellbeing outcomes such as affect and emotion (Frost et al., 2022; Kitson et al., 2018; Lee et al., 2022; Liu et al., 2025b; Riches et al., 2021, 2023, 2024; Syed Abdullah et al., 2021; J. Wang et al., 2022; Q. Wang et al., 2023). However, research to date lacks insight into the mechanisms driving these outcomes. This can be attributed to two key research limitations. Firstly, there is a swathe of different methodologies and VR designs present across studies, making comparison of their findings difficult (Frost et al., 2022; Kitson et al., 2018; Lee et al., 2022; Riches et al., 2021, 2023, 2024). Secondly, individual studies rarely include comparison or control conditions to examine how a specific, experimental VR environment is producing a change in outcomes. VR design elements such as stimuli, embedded tasks, framing, and instructions can be manipulated to encourage particular user–VR interactions, making it difficult to isolate the effect of any single component (e.g., Riches et al., 2021, 2023, 2024).

Within the positive technology framework, a meaningful distinction is whether a technology system is aimed at engaging the user in hedonic or eudaimonic wellbeing development. Hedonic wellbeing refers to the presence of pleasure and absence of pain in one’s life, generally characterised by high levels of subjective wellbeing (Diener, 2009; Fredrickson, 2001, 2004, 2013; Fredrickson et al., 2000; Waterman, 1993). Eudaimonic wellbeing refers to the presence of meaning, personal values, and interpersonal connections (Deci & Ryan, 2008; Ryan et al., 2008; Ryan & Deci, 2000a, 2000b; Ryff, 1989, 2013, 2018; Waterman, 1993; Waterman et al., 2008). Riva and Gaggioli (2015) proposed that positive technologies could be categorised by these two dimensions of wellbeing. Hedonic technologies induce positive and pleasant experiences, eudaimonic technologies facilitate self-actualisation and encourage introspection or personal growth, while social/interpersonal technologies support and improve social connectedness (Riva & Gaggioli, 2015). While hedonic and eudaimonic dimensions are described as conceptually distinct categories, in practice they frequently overlap and interact in lived experience (Henderson & Knight, 2012; Kashdan et al., 2008). The present study isolates them as independent variables for experimental purposes, rather than treating them as mutually exclusive in a broader sense. This distinction in terms of wellbeing related VR design, offers a fruitful route to exploring the underlying mechanisms. Applying this hedonic/eudaimonic distinction to the current literature of VR and wellbeing, a clear pattern emerges. There is a prevalence of research focused on hedonic VR applications. Comparatively, the theoretically supported potential of eudaimonic VR environments is underexplored. We propose that comparing hedonic and eudaimonic VR features will make an important contribution to understanding the mechanisms by which VR environments impact wellbeing outcomes. In the present study, this distinction is operationalised through two specific features: (1) the VR setting and (2) the level of personal meaning.

### 1.1. VR Setting: Virtual Nature and Hedonic Outcomes

While the hedonic benefits of real and non-VR forms of simulated nature exposure are well established (e.g., Bowler et al., 2010; Browning et al., 2020, 2023; Capaldi et al., 2015; McMahan & Estes, 2015; Shuda et al., 2020), applied VR studies have rarely compared nature to other settings directly when measuring state-based outcomes such as emotion and affect. Recent studies have begun to bridge this gap by directly comparing nature and urban VR environments (Chan et al., 2023; Li et al., 2021; Mostajeran et al., 2021; Schutte et al., 2017; Yu et al., 2018, 2020). However, results in this area have been mixed and inconsistent to date with some reporting comparable self-report and bio-feedback responses (Frost et al., 2022; Li et al., 2021; Mostajeran et al., 2021; Schebella et al., 2019; Yu et al., 2018, 2020). Conversely a number of studies report higher positive and lower negative wellbeing on self-report metrics including happiness, positive and negative affect, mood, perceived restorativeness, fatigue, or mood disturbance after experiencing VR nature, relative to VR urban environments (Chan et al., 2023; Mostajeran et al., 2021; Schebella et al., 2019; Schutte et al., 2017; X. Wang et al., 2022; Yu et al., 2018, 2020). Systematic reviews similarly report mixed effects of VR nature exposure on emotion and affect (Frost et al., 2022; Lee et al., 2022; Liu et al., 2025a, 2025b; Syed Abdullah et al., 2021), with inconsistency partly attributable to variation in study designs, exposure times and outcome measures. Despite inconsistency in effects across studies, taken as a whole, results trend towards VR nature environments having greater positive effects than urban environments on hedonic outcomes. Studies which introduce comparison conditions and present a simplified methodology, and VR design may clarify the contribution of VR nature exposure itself on hedonic outcomes.

### 1.2. VR and Eudaimonic Content: Presenting Meaningful Content

Compared to the body of research examining participants’ responses to hedonic VR environments, considerably less work has explored the effects of eudaimonic VR content on state-based outcomes such as emotion or affect. This research can broadly be grouped into two approaches. The first approach seeks to evaluate meaningful experiences which are facilitated by interacting with intentionally designed environments. For example, VR enrichment, for older adults, seeks to provide meaningful experiences such as VR travel that are not otherwise accessible (Holloway et al., 2024; Ke et al., 2025; Khirallah Abd El Fatah et al., 2024; Lin et al., 2018; Restout et al., 2023; Wei et al., 2025). Yet, the creation and examination of a meaningful experience or interaction with meaningful VR content is not the goal of these studies, rather, they focus on VR experiences as a tool for creating hedonic and subjective wellbeing supporting activities for an underserved population. Other studies aim to induce awe or self-transcendent states with vast natural phenomena or expansive abstract scenes (Barberia et al., 2018; Chirico et al., 2017, 2022, 2024; Chirico et al., 2018a, 2018b; Chirico & Gaggioli, 2019, 2023). The central research question in such work concerns whether VR can reproduce emotional and physiological profiles consistent with awe. Although awe is often described as a meaningful emotional experience, the environment and content are not personally meaningful per se; rather, they are constructed to evoke a state associated with eudaimonic experience. Moreover, the emphasis remains on demonstrating the successful induction of that state, rather than examining the broader psychological consequences of engaging with meaning through the medium of VR.

The second approach incorporates personal meaning into the user–VR interaction, to produce hedonic supporting experiences, such as reminiscence. For example, Fernandez-Alvarez et al. (2021) utilised Google Earth VR to immerse participants in locations associated with positive past experiences to enhance positive autobiographical recall. Baños et al. (2011, 2013, 2014) embedded prompts within relaxing nature environments to guide participants toward reminiscing on joyful or calming memories. In these cases, personally relevant content is central; however, it is utilised as a pathway to positive affect induction. The emphasis lies in improving mood or inducing positive emotional states, rather than isolating the psychological effects of interacting with personally meaningful VR content. Meaning, therefore, functions as a method for inducing a hedonic state, rather than the VR feature under investigation. In both approaches, meaningful content operates as one component of a broader research protocol, making it difficult to isolate its specific emotional consequences. Establishing how individuals respond emotionally to meaningful VR environments and experiences, which are not manipulated in their design or selection to be positive, negative or otherwise is a necessary first step in determining whether VR can serve as a platform for eudaimonic engagement.

### 1.3. The Present Study

The present study explores how two VR design features influence participants’ emotional responses: setting (nature vs. urban) and personal meaning (personally meaningful vs. non-personally meaningful). Nature is operationalised as a hedonic design feature, given its prominence in VR wellbeing research and its theorised association with positive affective outcomes. Urban environments serve as a comparison condition. Brief VR exposures of this duration have been shown to produce measurable changes in self-reported emotion in previous research (e.g., Chirico et al., 2017; Schutte et al., 2017). Unlike prior applied research, comparing the effect of exposure alone is the primary objective, and VR environments do not include tasks (e.g., breathing exercises) or extensive framing (e.g., try and enter a relaxed state) aimed at inducing a targeted state-based outcome. Differing from previous direct comparison studies, which utilise bespoke specifically designed nature and urban environments, the present study examines the influence of setting and personal meaning on state-based emotion responses using scenes presented through the same VR application.

Personal meaning is operationalised as a eudaimonic design feature. Unlike selecting a location based on aesthetics or familiarity, choosing a location based on personal meaning is intended to engage identity-relevant content. These are places associated with significant experiences, relationships, or values that contribute to one’s sense of self and continuity (Markus & Wurf, 1987; McAdams, 2001, 2003). It is this identity-relevance that distinguishes the manipulation as eudaimonic rather than simply hedonic or autobiographical. Meaning is central to the many different conceptualisations of eudaimonic wellbeing and so it is appropriate to utilise it to create an eudaimonic interaction with the VR environment (e.g., Deci & Ryan, 2008; Frost et al., 2022; Ryan & Deci, 2001; Ryff, 1989, 2013; Waterman, 1993, 2008). A theoretically grounded candidate for providing a VR–user interaction with meaning at its centre can be derived from identity and self-concept research. A coherent sense of self, encompassing past experiences, present identity, and anticipated future selves is central to psychological wellbeing, particularly eudaimonic aspects such as meaning, authenticity, and personal growth (Erikson, 1994; King et al., 2006; King & Hicks, 2009; McAdams, 2003; Orenstein & Kaur, 2026). Meaningful experiences frequently involve engagement with personally significant memories, values, or life narratives that reinforce continuity and purpose (Markus & Wurf, 1987; Sadeh & Karniol, 2012; Urminsky, 2017; Vignoles et al., 2011). Despite evidence that self-referential and identity-relevant content can influence emotional experience and wellbeing (Carr et al., 2021; Layous et al., 2013; Sheldon & Lyubomirsky, 2006; Sin & Lyubomirsky, 2009; Sokol & Serper, 2019), the potential for VR to present personally meaningful, identity-relevant environments as a standalone experiential stimulus has received limited empirical attention.

VR platforms such as Google Earth VR provide a practical means of operationalizing such content, allowing participants to immerse themselves in real-world locations selected for their personal meaning. Importantly, personally meaningful places represent a prompt that is meaningful but not directive in terms of being wholly positive or negative. Research on nostalgia and autobiographical memory suggests that engagement with personally significant content frequently elicits mixed emotional responses, encompassing both positive and negative affect (Routledge et al., 2008, 2011; Sedikides et al., 2008; Wildschut et al., 2006; Wildschut & Sedikides, 2023). The possibility that meaningful locations may evoke both positive and negative memories is, therefore, treated as a theoretical feature of eudaimonic engagement rather than a confound. Personally meaningful virtual locations, therefore, represent a theoretically grounded operationalisation of eudaimonic VR content, suitable for investigating how interaction with meaningful environments shapes emotional experience. In the present study, participants interact with VR simulations of real-world locations that were either self-selected for their personal meaning or selected by the experimenter. This manipulation is designed to isolate the emotional consequences of engaging with meaningful content without directing participants toward explicitly positive or negative selections. Of particular interest is whether meaningful environments would elicit not only elevated positive emotion, but also potentially increase negative emotion, consistent with theoretical accounts of eudaimonic engagement involving mixed affective experience.

Exploring setting and personal meaning in a single within-subjects design allows for the examination of their relative and interactive influence on positive and negative emotion outcomes. If VR environments function in line with wellbeing and positive technology theory, exposure to nature settings would be expected to increase positive emotions and reduce negative emotions relative to urban settings. Personally meaningful environments are expected to increase positive emotions and may also elevate negative emotions relative to non-meaningful environments. An interaction effect is predicted whereby nature-based, personally meaningful environments produce the most favourable overall emotional profile.

Emotional responses are conceptualised in line with the theory of constructed emotion (Barrett, 2006, 2017) which proposes that emotions are not fixed, biologically discrete entities, but are constructed through the appraisal of sensory input and internal physiological states based on prior experience. From this perspective, changes in emotional response following VR exposure reflect participants’ subjective interpretations of their interaction with the environment. Given the absence of definitive objective markers for discrete emotion categories (Barrett, 2006, 2017), self-report remains an appropriate and theoretically consistent method for assessing emotional experience. Accordingly, participants report their levels of positive (desire, happiness, relaxation) and negative (anger, disgust, fear, anxiety, sadness) emotions following each condition. A repeated-measures design further allows for within-person comparison across environmental conditions, minimising variability attributable to individual differences in emotional categorisation.

Study Aim: To examine the relative contribution of VR settings (nature vs. urban) and the level of personal meaning (personally meaningful vs. non-personally meaningful) to participants’ emotion responses. It should be noted that the present study does not directly measure eudaimonic constructs such as meaning in life or self-reflection. Rather, the personally meaningful location selection manipulation is employed as a theoretically grounded proxy for eudaimonic engagement, and the emotional responses it elicits are examined as indicative of such processes.

### 1.4. Hypotheses

The present study hypotheses were pre-registered on aspredicted.com (#: 90,392).

**Hypothesis** **1.** *The personal meaning of the environment will interact with the environment setting to influence the emotions reported by participants. Specifically, it is predicted that exposure to a nature-based, personally meaningful environment will produce the best (higher positive and lower negative) emotion outcomes*.

**Hypothesis** **2.** *Exposure to nature settings will result in higher positive emotions and lower negative emotions than exposure to urban settings*.

**Hypothesis** **3.** *Participants will report higher levels of positive emotions and lower levels of negative emotions following exposure to personally meaningful compared to non-personally meaningful conditions*.

## 2. Materials and Methods

### 2.1. Design

A 2 × 2 within-subjects exploratory experimental design was used to explore how environment setting and personal meaning influence participants’ self-reported emotion responses. Setting (nature vs. urban) and personal meaning (meaningful vs. non-meaningful) were the within-subjects variables. Each participant experienced four different conditions in virtual reality (see Section 2.5), and their emotion responses were recorded immediately after each exposure. The design and analysis plan for this study were pre-registered on the website aspredicted.org. (#: 90,392).

### 2.2. Participants

A g*power (V. 3.1.9.7) analysis for a study with a within-subject’s groups design, two independent variables and four conditions with a medium effect size (F = 0.25, α = 0.05, β = 0.2, *r* among RM = 0.50) indicated a minimum required sample size of twenty-four participants to test the interaction effect and thirty-four to test main effects. Participants over 18 years old were recruited with no exclusion criteria, using convenience sampling from a combination of social media posts (e.g., LinkedIn, Instagram), email lists, recruitment posters and lecture announcements in two universities in Dublin. In total, thirty-six participants completed the experiment. One participant was removed from the present analysis because they failed seven out of ten of the attention checks (see measures). The final sample contained 35 participants (18 female, 16 male, 1 undisclosed gender; age M = 28.14, SD = 9.614). All demographics were normally distributed except for age, which had a skewness (2.74) and kurtosis (7.85) outside the expected range. This was likely due to two participants with age above the sample mean (60+). Experience using VR technologies was low, with 27 participants (77.1%) reporting that they never used VR technologies (*n* = 13) or only used once before (*n* = 14). The remaining 22.9% of participants indicated using VR once a year (*n* = 5), once a month *(n* = 2) or daily (*n* = 1).

### 2.3. Materials

#### 2.3.1. Stimuli Presentation (Hardware)

Two Oculus Quest 2 VR headsets were used to run a Google Earth VR programme (GEVR; Google LLC, Mountain View, CA, USA; https://arvr.google.com/earth/; accessed on 31 May 2022). This was streamed to the headsets from two separate desktop computer systems. The first was a Dell XPS Desktop 8950 (Dell Technologies, Round Rock, TX, USA; 16 GB RAM) running a NVIDIA(R) GeForce RTX(TM) 3060 Ti 8 GB (LHR) graphics card (NVIDIA Corporation, Santa Clara, CA, USA). The second was a Lenovo Desktop (Lenovo Group Ltd., Beijing, China; 16 GB RAM) with an NVIDIA GTX1070 graphics card (NVIDIA Corporation, Santa Clara, CA, USA). Both systems met the minimum specifications required to run GEVR at full quality.

#### 2.3.2. Stimuli Presentation (Software)

**Figure 1:**
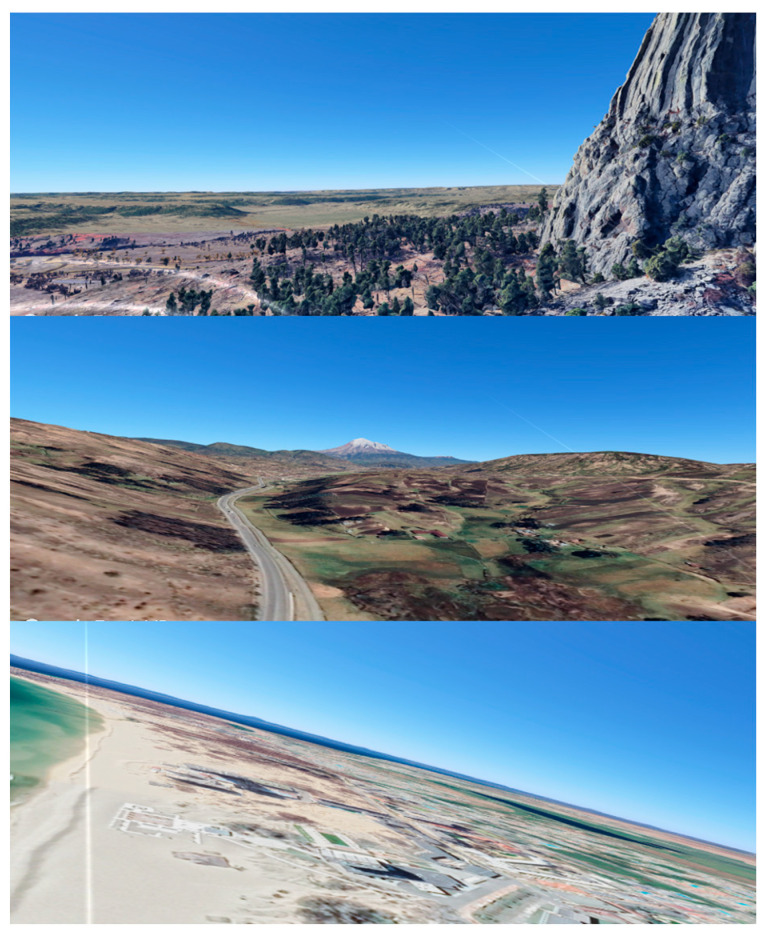
Google Earth VR nature locations (Condition 1). Note. From top to bottom—Wyoming Forest | Ecuadorian Mountains | Mediterranean Beach.

**Figure 2:**
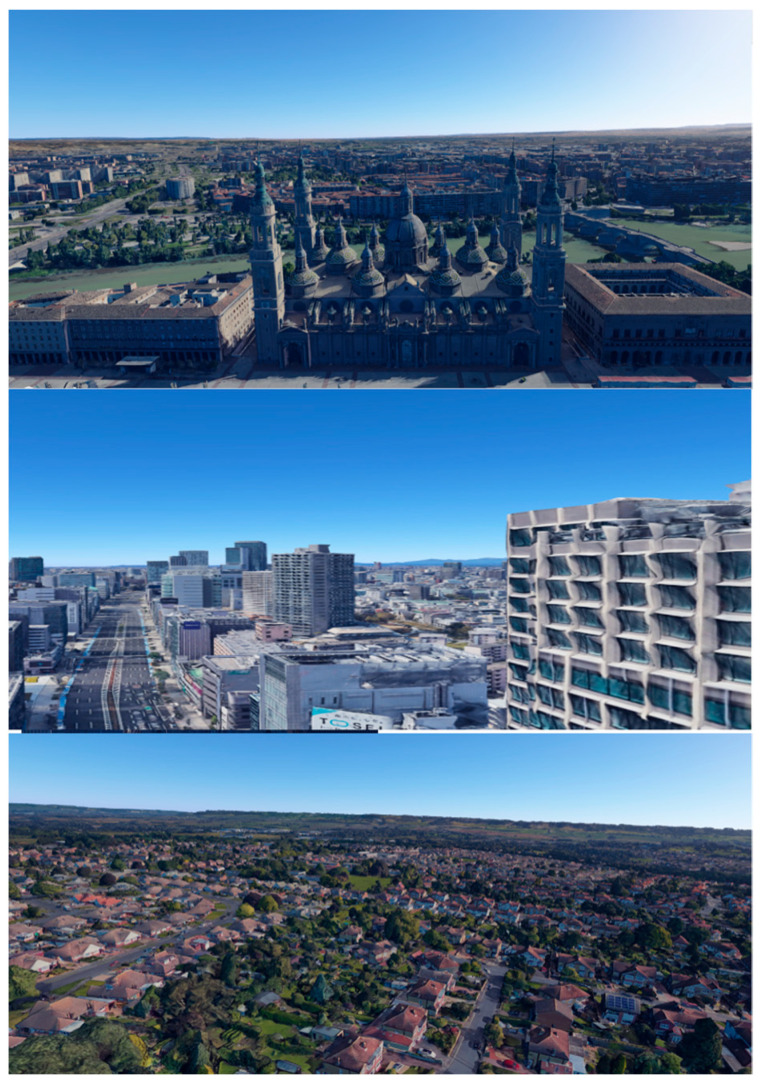
Google Earth VR urban locations (Condition 2). Note. From top to bottom—Spanish cathedral (landmark) | Tokyo (city) | suburban England (residential area).

Google Earth VR (GEVR) was used to present all conditions and stimuli throughout the course of this study. This is an open-world programme, based on 3D renders of the satellite images used in Google Earth desktop programme (GEVR; Google LLC, Mountain View, CA, USA; https://arvr.google.com/earth/; accessed on 31 May 2022). The programme allows participants to explore vast, immersive, virtual versions of real-world locations (see Figure 1 and Figure 2). The programme also has the same street view feature as the desktop version, so participants could travel through immersive, 360° representations of areas such as streets or parks. This feature uses real images from the Google Earth database. Combined with the immersive characteristics of VR systems, this gives a sensation approximate to travelling through the area as they would in the physical world (see Figure 1 and Figure 2). No sound accompanied the visual stimulus across all conditions. This design decision was made to focus the design on isolating setting and level of personal meaning as the manipulations of interest and limit the number of potentially confounding differences between conditions. However, this has a notable trade-off of potentially limiting the participants immersion and the level of ecological validity of the presented VR environments across the study conditions.

### 2.4. Measures

#### 2.4.1. Self-Reported Emotion State

The Discrete Emotions Questionnaire (DEQ) was used to measure participants’ positive and negative emotions at baseline and after each VR exposure. The DEQ is a thirty-six-item questionnaire which produces subscales for five negatively valanced (anger, disgust, fear, anxiety, sadness) and three positively valanced (happiness, relaxation, and desire) emotions and is suitable for detecting state-based changes in emotion (Harmon-Jones et al., 2016). Participants indicate the extent to which they felt a particular emotion on a 7-point Likert Scale from 1 (not at all) to 7 (an extreme amount). The internal consistency of all subscales is high (Cronbach’s α > 0.80; Harmon-Jones et al., 2016). As an exploratory analysis, this study was interested in whether participants’ positive and negative emotion profile changed in general. The aggregation of discrete emotion subscales into composite positive and negative scores is consistent with the theory of constructed emotion (Barrett, 2017), which treats valence as a fundamental dimension of emotional experience. For this reason, items for each of the five negative emotions subscales were totalled to form a Total Negative Emotions Subscale, which had good internal reliability at each administration of the DEQ (α ≥ 0.79). The same was performed for the three positive emotion subscales to form a Total Positive Emotions Subscale, all of which had good internal reliability (α ≥ 0.69).

#### 2.4.2. Attention Checks

In each administration of the DEQ, two attention check items were added. For example, the participant would see ‘attention check click moderately’. The one participant who failed multiple checks across the study was removed from the analysis.

### 2.5. Procedure

After consenting, participants answered a series of questions to collect general basic demographic information consisting of age, gender, education level and experience using VR (e.g., once a day, week, month), then the baseline version of the DEQ was completed.

Participants were fitted with the VR headset, then entered the GEVR programme and completed a short tutorial (5 min approx.) to help them adjust to the feeling of being in VR and teach them the GEVR controls. In all conditions, participants were asked to stay in the general vicinity of the selected location but were given freedom to navigate the environment and alternate between the free roam and street view options provided by GEVR. Participants then completed all conditions in a fully randomised order (not counter-balanced) while seated. Between each condition, participants exited GEVR and completed the DEQ, this served as a break from VR between conditions. Randomisation and breaks were intended to mitigate the risk of carry over effects between conditions. Finally, participants completed questions about their experience such as which condition was their favourite. Elements of the procedure specific to each condition are described below.

#### 2.5.1. Condition 1: Non-Personally Meaningful Nature (NPM-Nature)

Participants were presented with three nature locations to choose from; a beach, a forest, and a mountain range (see Figure 1). These three locations were chosen based on a high likelihood of being novel for participants and intended to represent a diverse range of nature settings. Participants were asked to *‘choose the one you would most like to visit now’*. After exploring this location, they removed the VR headset and were presented with the options again and asked, *‘of the two you are yet to visit, please choose the one you would most like to visit now’*. They then explored the final environment. Participants spent 2 min in each location, for a total of 6 min of VR exposure in total. The element of choice was deliberately introduced to partially mirror the self-selection freedom provided in the personally meaningful conditions, while maintaining experimenter control over the location options.

#### 2.5.2. Condition 2: Non-Personally Meaningful Urban (NPM-Urban)

The procedure for this condition was identical to Condition 1, except the options given to participants were a large city, a residential area, and an urban landmark (see Figure 2). These three locations were chosen based on a high likelihood of being novel for participants and intended to represent a diverse range of urban settings. As in Condition 1, an element of choice was incorporated for the same reason.

#### 2.5.3. Condition 3: Personally Meaningful Nature (PM-Nature)

Participants were given the instructions *‘Please think of a location in a nature setting that has meaning for you and that you would like to visit now. For example, a place you visit regularly, have travelled to or want to go to’.* They then travelled to this location and spent 2 min exploring it. This process was repeated two more times with participants selecting different meaningful locations for a total of 6 min of exposure to meaningful locations. Headsets were not removed in between locations as the process of choosing a new location in this condition was performed through GEVR.

#### 2.5.4. Condition 4: Personally Meaningful Urban (PM-Urban)

The procedure for this condition was identical to Condition 3, except participants were asked three times to *‘Please think of a location in an urban setting that has meaning for you and that you would like to visit now. For example, a place you visit regularly, have travelled to or want to go to’*.

### 2.6. Ethical Approval

This study received ethical approval from Dublin City University Research Ethics Committee and an exemption from full ethics review from the UCD Research Ethics Committee. Participation in the present study was fully voluntary; opt-in and no incentives were provided for participating.

### 2.7. Data Analysis

IBM SPSS Statistics (Version 29) was used to perform all analyses presented below. Prior to analysis, normality of residuals and homogeneity of variance were checked. DEQ subscales were calculated, and items checked for missing data. Baseline DEQ scores were subtracted from post-exposure scores for each subscale to allow assessment of change scores across conditions. The internal reliability of all DEQ subscales were checked. Self-reported emotion response data was analysed using a series of 2 × 2 within-subjects factorial ANOVA with setting (nature and urban) and personal meaning (personally meaningful and non-personally meaningful) as the independent variables. Eight factorial ANOVA were run to establish the differences between participants’ responses on each of the eight distinct emotion subscales of the DEQ. Additionally, two exploratory ANOVA using the total positive emotion and total negative emotion scales derived from the DEQ were run to examine differences in overall positive and negative emotion profiles following exposure to each condition. To control for Type I error across multiple comparisons, Bonferroni correction was applied within each family of tests corresponding to each hypothesis, with 10 tests per family and a corrected significance threshold of α ≤ 0.005. Order effects were not formally examined in the present study, but a fully randomised condition order was employed to distribute any such effects equally across conditions.

## 3. Results

### 3.1. Data Management

#### 3.1.1. Missing Data and Data Cleaning

A missing completely at random analysis was conducted for each DEQ item score recorded. Of a total 5950 DEQ values, 21 were missing completely at random. Thirteen participants had missing data for at least one of the DEQ items. No participant had missing data for more than one item on a single subscale and no single item on the DEQ had more than one missing value. Given the low frequency and the randomness of the missing data values, mean imputation was employed as the data management technique. The internal reliability of all DEQ subscales were checked. Mean DEQ scores were calculated for participants on each of the 8 DEQ subscales (anger, disgust, fear, anxiety, sadness, happiness, relaxation, and desire), total positive emotions and total negative emotions at baseline and following each condition (1–4), from which change scores were derived.

#### 3.1.2. Scale Reliability

*DEQ Internal reliability:* Internal reliability analyses were run for each subscale (anger, disgust, fear, anxiety, sadness, happiness, relaxation, desire), total positive emotion, and total negative emotion for each condition (baseline, NPM-Nature, NPM-Urban, PM-Nature and PM-Urban) using Cronbach’s Alpha. A total of fifty reliability analyses were run. The majority of the scales and subscales had high internal reliability (40/50) with a small number having moderate (5/50) and low (5/50) reliability (see Table 1). Most reliabilities had an alpha value of ≥0.7 and, therefore, were within the acceptable range for Cronbach’s alpha (Tavakol & Dennick, 2011). Four of the subscales had an alpha value of 0.6–0.69, however they fell within the acceptable range for scales with less than ten items (Loewenthal & Lewis, 2001). Five subscales had a low reliability (α < 0.6) clustered in the fear (2), sadness (2), and disgust (1), and all instances of low reliability were in the baseline condition or the non-personally meaningful nature condition. This is noted because these conditions had the lowest scores in these constructs and so the low alpha may result from floor effects, rather than construct instability. Nevertheless, results involving these subscales should be interpreted with caution (see Table 1). Notably, no subscale had low reliability across all conditions, suggesting the measure maintained adequate integrity overall.

### 3.2. Hypothesis Testing

A series of 2 × 2 within-subjects ANOVA were run to test the study hypotheses. Statistics for DEQ negative emotion subscales and total negative emotion are reported in Table 2, while those for positive emotion subscales and total positive emotion are reported in Table 3. The full summary statistics (mean, confidence intervals and standard error) for each subscale are reported by condition in Table 4.

Hypothesis 1 predicted a significant interaction between VR environments’ setting and level of personal meaning such that participants would report different profiles of discrete emotions across conditions. It was predicted that personally meaningful nature environments would produce higher positive and lower negative emotion outcomes than other conditions. No significant interaction effects were observed on participants’ self-reported positive discrete emotion subscale scores (see Table 3) or negative discrete emotion subscale scores (see Table 2). Additionally, no interaction effect was observed to influence total positive or total negative emotion scores. Hypothesis 1 was not supported by the results of the present study.

Hypothesis 2 predicted that, regardless of the level of personal meaning, nature-based VR locations would result in higher levels of self-reported positive emotions and lower levels of self-reported negative emotions than urban-based VR locations. There was no main effect of setting on total positive emotion (see Table 3) nor total negative emotion (see Table 2) scores. There was also no significant effect of setting on the discrete emotion subscales. Thus Hypothesis 2 was not supported by these results.

Hypothesis 3 predicted that, regardless of their setting, exposure to personally meaningful locations in VR would result in higher levels of self-reported positive emotions and lower levels of self-reported negative emotions compared to non-personally meaningful (experimenter-selected) locations. Total negative emotion scores and discrete emotion subscale scores did not differ based on the level of personal meaning, except for sadness (see Table 2). The main effect of sadness had a large effect size (see Table 2) and mean scores indicated an increase in sadness following exposure to meaningful environments compared to experimenter-selected non-meaningful ones (see Table 5). Main effects of personal meaning were observed for the total positive emotion and desire subscales but not the happiness or relaxation subscales (see Table 3). The effect size was large for both desire and total positive emotion scores, with mean scores indicating that both variables increased following exposure to personally meaningful compared to experimenter-selected non-meaningful VR environments (see Table 5). Hypothesis 3 is partially supported by these results. Total positive emotion and desire increased in line with the hypothesis, but the increase in sadness is contrary.

## 4. Discussion

The present study sought to explore how exposure to VR environments influenced participants’ emotional responses, with a specific focus on isolating two theoretically relevant factors: environmental setting and level of personal meaning. By adopting a relatively simplified methodology and VR design, this study aimed to examine the extent to which emotional change could be attributed to the characteristics of the environment (nature vs. urban), the personal significance of the VR environment, or their interaction, rather than to an accompanying task or embedded intervention.

Hypothesis 1 predicted that an interaction would occur between the level of personal meaning associated with an environment and its setting. Specifically, it was predicted that personally meaningful environments in a nature setting would demonstrate the greatest influence on self-reported emotion responses. No significant relationship between the two independent variables influenced participants’ positive or negative emotion responses. Hypothesis 2 predicted that VR nature settings would result in greater increases in positive and decreases in negative emotions than urban settings. No significant effects of setting were observed on any emotion outcome. Total positive and total negative emotion scores did not differ following exposure to nature compared to urban environments. Hypothesis 3 predicted that exposure to personally meaningful VR environments would produce greater increases in positive emotion and greater decreases in negative emotion than exposure to non-meaningful environments. Results showed that personally meaningful environments were associated with higher total positive emotion scores, higher desire scores, and with increased sadness.

The findings reveal interesting patterns about the influence of VR setting and level of personal meaning on participants’ emotion responses. Contrary to hypothesis 1, no interaction between setting and personal meaning was observed. Yet, main effects were observed for personal meaning. This suggests that level of meaning, as a feature of the VR design, exerted an independent effect that partially aligned with hypothesis 3. Given the exploratory nature of this study, interpretations below are an attempt to understand the present results but can be considered as interesting points on which future research can seek to build, rather than definitive statements of the comparison between hedonic and eudaimonic features of VR environments.

### 4.1. The Absence of an Interaction Effect on Emotion Outcomes

Hypothesis 1 predicted an interaction effect based on prior research showing that both exposure to nature and engagement with personally meaningful content are associated with positive hedonic outcomes. However, a key limitation of that literature is that hedonic and eudaimonic features of VR environments have rarely been manipulated independently, for example, personal meaning has typically been embedded within hedonic environments as a tool for inducing positive affect. For researchers interested in the underlying mechanisms of change, it is difficult to determine whether observed wellbeing effects were genuinely interactive or simply co-occurring. The present study addressed this directly by manipulating setting and personal meaning as independent variables within the same design. No interaction was found, indicating that when separated, these variables exert independent rather than combined effects on emotion outcomes. This meaningful contribution suggests that the interactive effects implied by previous research may have been a product of how those studies were designed, rather than a genuine property of the variables themselves.

### 4.2. The Absence of Setting Effects on Emotion Outcomes

Environmental setting was not associated with any significant effects. This finding is consistent with a broader pattern of mixed and inconsistent results in the VR nature literature (Chan et al., 2023; Li et al., 2021; Mostajeran et al., 2021; Schutte et al., 2017; Yu et al., 2018, 2020) and adds to this pattern using a broad measure of discrete emotion. More broadly, the absence of setting effects, cautiously interpreted within the constraints of the present study, suggests that the hedonic benefits often associated with VR nature exposure may not be automatic and may be augmented with additional activities or framing (Riches et al., 2021, 2023, 2024; Velana et al., 2022). It can be noted in Table 2 and Table 3 that some emotion effects did not survive the statistical correction for multiple tests. Fear and desire showed medium effect sizes in the expected directions and may warrant further investigation in future research with larger samples.

### 4.3. The Mixed Emotional Pattern of Personally Meaningful VR Environments

The integration of personal meaning into VR environments was associated with changes in emotion outcomes in the present study. Specifically, exposure to personally meaningful locations was characterised by an increase in total positive emotion and in desire. It was also characterised by an increase in sadness. Taken together, it appears that visiting personally meaningful locations in VR evoked a mixed emotional response from participants. These findings are consistent with literature on engagement with meaningful personal content, including nostalgia and reminiscence, which are often associated with feelings of warmth, longing, and wistfulness for a point in time and a mixed emotional response (Routledge et al., 2008, 2011; Sedikides et al., 2008; Wildschut et al., 2006). Despite their mixed-emotional nature, previous research suggests that these personally meaningful experiences can be utilised as positive wellbeing resources (Brown & Humphreys, 2002; Iyer & Jetten, 2011; Routledge et al., 2008, 2011; Sedikides et al., 2008; Wildschut et al., 2006; Wildschut & Sedikides, 2023). One speculative interpretation of the desire finding concerns its meaning in this specific context. While desire is treated as a positive emotion in the DEQ (Morgan & Farsides, 2009), it is possible that in the context of briefly visiting personally meaningful locations through GEVR, it reflects something closer to wistfulness or yearning rather than purely positive wanting. The mixed emotional response observed in the present study suggests that participants found the content personally meaningful.

The results of the present study suggest the level of personal meaning present in the VR environments had a more complex influence on changes in participants’ emotions than environmental setting. In contrast to previous studies utilising personally meaningful content or seeking to create meaningful interactions with VR, this effect was produced using personal content, integrated into a non-directive interaction with the VR. In other words, the experimental manipulations in this study did not attempt to frame the VR environment in a positive or negative way, so it is likely that observed changes were due to participants’ personal attachments to the visited locations. As such, the present findings indicate that a brief, personalised VR exposure could elicit emotionally complex responses consistent with engagement with meaningful content. Within this context, VR researchers and designers should consider the implications of utilising autobiographical content that may elicit unintended or unexpected emotional responses. The potential for personally meaningful content to evoke complex emotional reactions should be carefully considered, particularly in non-clinical settings where appropriate support structures may not be in place.

In summary, there was an absence of both an interaction effect and setting effects on emotion outcomes, while a pattern which suggests a mixed emotional response to personally meaningful VR environments was observed. Taking the results together, the findings contribute to previous debates around the conceptualisation of hedonic and eudaimonic processes as separate but related dimensions of wellbeing (Henderson & Knight, 2012; Kashdan et al., 2008; Ryff, 2013). Henderson and Knight (2012) propose that hedonic and eudaimonic pursuits are distinct, operating through different psychological mechanisms, but are complementary in their contribution to holistic wellbeing. The present findings make a direct contribution to this position. They suggest that in this exploratory design, personal meaning, as a eudaimonic feature, exerted independent effects on emotional outcomes, while nature as a hedonic feature did not. The findings align with the position that these dimensions may operate through distinct psychological pathways that require different conditions to activate.

### 4.4. Strengths and Limitations

The methodological qualities of this exploratory study must be acknowledged and the findings caveated within this context. The sample size was sufficient to detect medium effects but may have been insufficient to detect smaller effects. This limitation is compounded by the application of multiple comparison corrections. For this reason, we report effect sizes for non-significant findings that may serve as useful indicators for future research with larger samples.

The use of Google Earth VR (GEVR) allowed nature and urban settings to be presented to participants, through the same VR platform, controlling technical factors such as the quality and rendering of visual stimuli. GEVR also facilitated the manipulation of personal meaning in the present study by presenting simulated versions of real-world locations. Mental imagery and visualisation are cognitively demanding processes which vary across individuals in terms of vividness and controllability (Cooley et al., 2013; Pearson, 2019; Williams & Cumming, 2012). GEVR minimised these individual differences in mental imagery as a factor that may influence the quality of engagement with meaningful content, and participants’ emotion responses to that content in the present study. The main limitation which came from the use of GEVR is the variation in the access to Google Street View which may have reduced comparability across heterogeneous conditions. In particular, nature settings are often remote, leading to limited travel while in street view compared to urban areas which generally allow full exploration. Differences in navigability and interactivity between settings may have influenced the depth or quality of engagement and are noted as an important limitation of this exploratory study. As such, equivalence between urban and nature conditions could not be guaranteed, and comparative setting effects should be interpreted cautiously. The study design made considerations for this by having participants visit three different locations so that a particularly limited location would not dominate their overall experience of the condition. Measures were taken after all three locations had been experienced, further reducing the impact of any one environment delivering a limited experience.

Additionally, it should be acknowledged that the study design did not include a way to measure the amount of meaning derived from each condition. For example, non-meaningful environments may have been experienced as meaningful or connected to personal autobiographical memories by the participants, which was not a factor controlled for by the authors. Related to this, it should be acknowledged that the absence of an exclusion criteria in the present study introduces a potentially confounding factor. For example, changes in emotion outcomes could reflect differences in mental health factors or negative responses to personal locations. However, participants were fully informed prior to consenting that they would be selecting and visiting real world locations in GEVR, these locations were self-selected and participants were informed before and during the experiment that they could withdraw or pause the study at any time in the case of discomfort or issues, limiting adverse user–VR experiences. Nevertheless, it remains a possibility that unscreened factors influenced emotional responses.

The way in which meaning was operationalised was a strength of the present study. By allowing participants to self-select environments, based on their meaningfulness, the study design ensured participants interacted with meaningful, personally relevant stimuli without positive or negative framing. However, while the observed pattern of emotional responses suggests that experiences such as nostalgia or catharsis may have been elicited, the present study did not capture information on what specifically made these locations meaningful to participants. As such, future research is needed to examine the content and personal significance of these experiences in more detail. A related limitation is the measurement approach. The Discrete Emotions Questionnaire (DEQ) is appropriate for measuring shifts in positive and negative emotion states but is not designed to assess more complex mixed emotional experiences. Engaging with personally meaningful content can evoke complex emotion states such as awe, nostalgia, or yearning. Though the pattern of discrete emotions observed in the present study suggests such experiences, the measurement approach was not designed to assess these more complex states directly. Additionally, at baseline, participants reported low scores on some negative emotions in the DEQ. Consequently, where participants had a close to minimum score at baseline, the DEQ was likely not sensitive enough to detect reductions in these negative emotions if they occurred across conditions. This floor effect likely contributed to the low reliability observed in certain subscale administrations (fear, sadness and disgust) and results involving these variables must be interpreted with caution. The present study relied solely on self-report measures of emotion. The inclusion of physiological or behavioural measures in future research would provide convergent evidence for the emotional responses reported here.

The novelty of interacting with VR for participants with low levels of prior experience may be considered a limitation (Miguel-Alonso et al., 2024). However, the use of a within-subjects design, where all four conditions were experienced by all participants within the same session and the use of the tutorial period before beginning, means that any novelty effects would have been distributed equally across conditions, minimising their potential to differentially influence outcomes. Finally, it should be noted that VR experiences vary considerably in their level of physical and cognitive engagement. The present study examined passive visual exposure to VR environments. Other VR applications, such as those used in therapeutic contexts, may engage participants through active social scenarios, structured tasks, or physical interaction demands that operate through distinct pathways (Mulvaney et al., 2026; Riches et al., 2021). While the limitations outlined above constrain the precision with which observed effects can be attributed to specific mechanisms of the user–VR interaction, they do not undermine the central contribution of the study. By systematically isolating environmental setting and level of personal meaning within a minimally framed VR exposure, the present study enabled a more mechanism-focused examination of VR–user interactions, which demonstrated distinct effects (or non-effects) on emotion outcomes. Within these parameters, the findings represent a constructive step toward clarifying how specific components of VR environments relate to emotion outcomes.

### 4.5. Future Research

Beyond comparative design, further work is needed to better understand how and why personally meaningful VR environments influence emotional responses. The present study demonstrated that self-selected meaningful locations were associated with a more pronounced change in both overall positive emotion and a more complex pattern of discrete emotion changes than nature environments. However, investigating if and how the participant’s experience of engaging with this content was meaningful is an interesting prospect for future research. A more direct examination of the characteristics of participants’ selected locations, including their relationship to autobiographical memory, identity, or future aspirations may also be of interest. Investigating this would allow researchers to move beyond identifying that personal meaning has an influence, toward understanding how it operates within VR–user interactions.

More broadly, future research should seek to examine the contribution of different elements of VR design and user experience to wellbeing outcomes. Rather than assuming that one type of environment, activity, or framing is universally optimal, investigation should focus on identifying components of the user–VR interaction that are most strongly associated with psychological aims. Nature-based environments may be well-suited to relaxation or affect regulation goals, while personally meaningful environments may be more appropriate for reflective, identity-relevant, or emotionally complex experiences for example. Systematic comparison setting, meaningful content and other aspects of VR environments’ design will help establish a clearer framework for matching VR design features to intended wellbeing outcomes.

## 5. Conclusions

The present study investigated whether interacting with VR environments that varied in setting and level of personal meaning influenced participants’ self-reported emotion responses. Results indicated that setting did not significantly influence any emotion outcomes while total positive emotion, desire, and sadness were all significantly influenced by personal meaning. These findings suggest that participants’ personal connection to a location had an influence on emotion outcomes while its setting did not. Personally meaningful VR environments elicited a pattern of mixed emotion, theoretically consistent with literature on eudaimonic engagement and personally meaningful experiences such as nostalgia, though research specifically investigating this topic is required. This study addressed a gap in the literature where VR research has often prioritised outcome change over examination of the underlying mechanisms that influence such changes. By isolating environmental setting and personal meaning within a minimally manipulated VR experience, the present design demonstrated that distinct components of VR experience can be experimentally examined. The methodological approach of this exploratory study provides a starting point for future research seeking to understand how specific features of VR environments contribute to wellbeing outcomes, rather than solely focusing on whether such outcomes occur. The findings further suggest personalising VR environments and linking them with eudaimonic concepts, as represented by personal meaning in the present study, represents an interesting avenue for future research into the use of VR to engage participants in wellbeing associated activities and interventions.

## Figures and Tables

**Table 1:** DEQ reliability scores.

Subscale	Baseline α	NPM-Nature α	NPM-Urban α	PM-Nature α	PM-Urban α
Happiness	0.69	0.83	0.82	0.88	0.88
Relaxation	0.78	0.70	0.87	0.86	0.89
Desire	0.78	0.81	0.84	0.73	0.81
Total Positive Affect	0.77	0.85	0.91	0.84	0.83
Anxiety	0.85	0.71	0.81	0.81	0.78
Anger	0.76	0.81	0.8	0.77	0.49
Disgust	0.5	0.78	0.6	0.74	0.71
Fear	0.34	0.45	0.91	0.79	0.75
Sadness	0.55	0.57	0.60	0.73	0.62
Total Negative Affect	0.68	0.82	0.87	0.85	0.77

Note. Low: *α* < 0.50; Moderate: *α* = 0.50–0.69; High = *α* > 0.70.

**Table 2:** Main and interaction effects for DEQ negative subscales and total negative emotions.

Measure	Effect	*F* (1, 34)	*p*	*η* ^2^_*p*_
Anger	Meaning	1.09	0.303	0.031
	Setting	1.09	0.239	0.041
	Interaction	0.11	0.707	0.004
Disgust	Meaning	3.27	0.079	0.088
	Setting	0.40	0.532	0.012
	Interaction	1.22	0.277	0.035
Fear	Meaning	4.00	0.053	0.105
	Setting	4.62	0.039	0.120
	Interaction	2.269	0.141	0.063
Anxiety	Meaning	1.28	0.266	0.036
	Setting	2.01	0.165	0.056
	Interaction	1.28	0.266	0.036
Sadness	Meaning	8.89	0.005 *	**0.207**
	Setting	0.54	0.466	0.016
	Interaction	0.06	0.8	0.002
Total Negative Emotions	Meaning	0.18	0.67	0.005
	Setting	3.29	0.079	0.088
	Interaction	0.31	0.582	0.009

Note. * = significant at the corrected *p* ≤ 0.005 level| Effect Size Parameters (Cohen, 1988) Low = 0.01 | Medium = 0.059 | High = 0.16.

**Table 3:** Main and interaction effects for DEQ positive subscales and total positive emotions.

Measure	Effect	*F* (1, 34)	*p*	*η* ^2^_*p*_
Desire	Meaning	10.87	0.002 *	**0.242**
	Setting	5.37	0.027	0.136
	Interaction	3.69	0.063	0.098
Relaxation	Meaning	1.71	0.2	0.048
	Setting	0.18	0.67	0.005
	Interaction	1.04	0.315	0.03
Happiness	Meaning	4.58	0.04	0.119
	Setting	0.555	0.462	0.016
	Interaction	1.27	0.267	0.036
Total Positive Emotions	Meaning	10.17	0.003 *	**0.23**
	Setting	1.74	0.196	0.049
	Interaction	3.71	0.062	0.098

Note. * = significant at the corrected *p* ≤ 0.005 level| Effect Size Parameters (Cohen, 1988) Low = 0.01 | Medium = 0.059 | High = 0.16.

**Table 4:** Estimated marginal means for DEQ subscales by condition.

Subscale	Condition	Mean	95% CI [LL, UL]	SE
Sadness	NPM-Nature	−0.043	−0.165, 0.079	0.06
	NPM-Urban	0.001	−0.091, 0.091	0.045
	PM-Nature	0.157	−0.029, -0.344	0.092
	PM-Urban	0.179	0.002, 0.355	0.087
Disgust	NPM-Nature	0.11	0.013, 0.206	0.048
	NPM-Urban	0.171	0.070, 0.273	0.05
	PM-Nature	0.079	−0.019, 0.176	0.048
	PM-Urban	0.064	−0.036, 0.165	0.05
Anger	NPM-Nature	−0.014	−0.097, 0.069	0.041
	NPM-Urban	0.007	−0.108, 0.122	0.057
	PM-Nature	0.001	−0.093, 0.093	0.046
	PM-Urban	0.043	−0.047, 0.132	0.044
Fear	NPM-Nature	−0.093	−0.186, 0.001	0.046
	NPM-Urban	0.001	−0.128, 0.128	0.063
	PM-Nature	−0.114	−0.206, -0.23	0.045
	PM-Urban	−0.086	−0.185, 0.014	0.049
Anxiety	NPM-Nature	−0.464	−0.698, −0.230	0.115
	NPM-Urban	−0.407	−0.666, −0.149	0.127
	PM-Nature	−0.524	−0.763, −0.284	0.118
	PM-Urban	−0.407	−0.666, −0.149	0.127
Total Negative Emotions	NPM-Nature	−0.101	−0.171, −0.031	0.035
	NPM-Urban	−0.046	−0.143, 0.051	0.048
	PM-Nature	−0.081	−0.152, −0.009	0.035
	PM-Urban	−0.047	−0.116, 0.022	0.034
Relaxation	NPM-Nature	−0.112	−0.464, 0.240	0.173
	NPM-Urban	−0.264	−0.684, 0.155	0.206
	PM-Nature	−0.067	−0.439, 0.306	0.183
	PM-Urban	−0.007	−0.328, 0.314	0.158
Happiness	NPM-Nature	0.321	−0.044, 0.687	0.18
	NPM-Urban	0.274	−0.129, 0.676	0.198
	PM-Nature	0.498	0.117, 0.878	0.187
	PM-Urban	0.664	0.285, 1.043	0.187
Desire	NPM-Nature	0.443	0.017, 0.869	0.21
	NPM-Urban	0.436	0.014, 0.857	0.207
	PM-Nature	0.707	0.270, 1.145	0.215
	PM-Urban	1.112	0.687, 1.537	0.209
Total Positive Emotions	NPM-Nature	0.214	−0.043, 0.471	0.127
	NPM-Urban	0.147	−0.136, 0.431	0.139
	PM-Nature	0.378	0.082, 0.674	0.145
	PM-Urban	0.6	0.353, 0.847	0.122

**Table 5:** Estimated marginal mean scores for significant main effects.

Main Effect	Subscale	PM			NPM		
Personal Meaning		Mean	95% CI [LL, UL]	SE	Mean	95% CI [LL, UL]	SE
	Sadness	0.168	0.003, 0.333	0.081	−0.021	−0.12, 0.077	0.048
	Desire	0.910	0.5, 1.319	0.202	0.439	0.041, 0.838	0.196
	Total Positive Emotions	0.489	0.238, 0.74	0.124	0.181	−0.076, 0.437	0.126

## Data Availability

The data presented in this study are available on request from the corresponding author.
